# Metabolomic Signatures of Scarff–Bloom–Richardson (SBR) Grade in Non-Metastatic Breast Cancer

**DOI:** 10.3390/cancers15071941

**Published:** 2023-03-23

**Authors:** Caroline Bailleux, David Chardin, Jocelyn Gal, Jean-Marie Guigonis, Sabine Lindenthal, Fanny Graslin, Laurent Arnould, Alexandre Cagnard, Jean-Marc Ferrero, Olivier Humbert, Thierry Pourcher

**Affiliations:** 1 Laboratory Transporter in Imaging and Radiotherapy in Oncology (TIRO), Direction de la Recherche Fondamentale (DRF), Institut des Sciences du Vivant Fréderic Joliot, Commissariat à l’Energie Atomique et aux Énergies Alternatives (CEA), Université Côte d’Azur (UCA), 06100 Nice, France; 2 Medical Oncology Department, Centre Antoine Lacassagne, University Côte d’Azur, 06189 Nice, France; 3 Department of Nuclear Medicine, Antoine Lacassagne Centre, 06189 Nice, France; 4 Department of Epidemiology and Biostatistics, Antoine Lacassagne Centre, University of Côte d’Azur, 06189 Nice, France; 5 Department of Tumour Biology and Pathology, Georges-François Leclerc Centre, 21079 Dijon, France; 6 Cenre de Ressources Biologiques (CRB) Ferdinand Cabanne, 21000 Dijon, France

**Keywords:** metabolomic signature, breast cancer, SBR grade, immunosuppression

## Abstract

**Simple Summary:**

Breast cancer is a heterogeneous disease with multiple biological, molecular, and histological subtypes. Several metabolomics studies have been performed on breast cancer cells highlighting their metabolic heterogeneity with a potential impact on the efficiency of personalized therapies. In our study, we performed an untargeted metabolomic analysis of breast cancer tumors and identified a metabolic signature for high-grade invasive tumors. AUCs for both the training set and validation set were above 0.88. This result indicates that the model can distinguish high-grade and low-grade tumors with a probability of almost 90%. We also identified several biomarkers of tumor aggressiveness, such as N1,N12-diacetylspermine and tryptophan catabolites, both of which are involved in the inhibition of the immune response. Our study thus provides new insights into the biological mechanisms underlying tumor aggressiveness. Furthermore, the identified biomarkers will enable the development of new strategies for better selection of patients in different immune therapy clinical trials, and thus, for better patient management. All these findings are discussed in relation to the latest publications in the field.

**Abstract:**

Purpose: Identification of metabolomic biomarkers of high SBR grade in non-metastatic breast cancer. Methods: This retrospective bicentric metabolomic analysis included a training set (*n* = 51) and a validation set (*n* = 49) of breast cancer tumors, all classified as high-grade (grade III) or low-grade (grade I–II). Metabolomes of tissue samples were studied by liquid chromatography coupled with mass spectrometry. Results: A molecular signature of the top 12 metabolites was identified from a database of 602 frequently predicted metabolites. Partial least squares discriminant analyses showed that accuracies were 0.81 and 0.82, the R2 scores were 0.57 and 0.55, and the Q2 scores were 0.44431 and 0.40147 for the training set and validation set, respectively; areas under the curve for the Receiver Operating Characteristic Curve were 0.882 and 0.886. The most relevant metabolite was diacetylspermine. Metabolite set enrichment analyses and metabolic pathway analyses highlighted the tryptophan metabolism pathway, but the concentration of individual metabolites varied between tumor samples. Conclusions: This study indicates that high-grade invasive tumors are related to diacetylspermine and tryptophan metabolism, both involved in the inhibition of the immune response. Targeting these pathways could restore anti-tumor immunity and have a synergistic effect with immunotherapy. Recent studies could not demonstrate the effectiveness of this strategy, but the use of theragnostic metabolomic signatures should allow better selection of patients.

## 1. Introduction

Breast cancer (BC) is a heterogeneous disease that includes several biological, molecular, and histological subtypes. Targeted and non-targeted metabolomics are promising approaches in the field of personalized medicine because they relate to the patient’s phenotype as closely as possible [1]. The targeted approach aims to identify a pathway or metabolite of interest based on a previously identified relationship. The untargeted approach seeks to identify and quantify as many metabolites as possible in a sample. Appropriate statistical analyses are then performed to determine which metabolites differ between the sample groups. Metabolite production changes when healthy cells turn into tumor cells with altered metabolism. This leads to metabolomic signatures that can reveal the presence of cancer cells with a specific cell behavior [2].

The study of metabolites in cancer can provide insights into how impaired metabolism can trigger proliferation, angiogenesis, and epithelial-mesenchymal transition (EMT) [3,4]. Because cancer cells have a sustained rate of growth and proliferation that requires a constant supply of metabolic precursors, significant changes in cell metabolism occur [5]. Metabolic reprogramming of cells and adjacent stroma is a key step in cancer development. The current biological model of carcinogenesis highlights various pathways for this process, such as escape from mechanisms involved in cell growth suppression, resistance to cell death, genomic instability and mutations, replication of immortalized cells, induction of metastasis capacity, tumor-induced inflammation, and immune system escape [6,7]. Several metabolomics studies have been performed with breast cancer cells [8,9,10]. For example, Gong et al. investigated metabolic dysregulation in Triple Negative Breast Cancers (TNBCs) using a multi-omics database. They classified TNBC samples into three heterogeneous metabolic-pathway-based subtypes (lipogenic, glycolytic or mixed) with distinct prognoses, molecular subtype distributions, genomic alterations, and distinct responses to personalized therapies targeting specific metabolic profiles [11]. To our knowledge, there is no publication reporting on studies that have specifically focused on the metabolomics of high-grade tumors.

Alterations in the metabolome can also be used as a potential indicator of breast cancer aggressiveness [12]. For example, metabolites of energy-generating metabolic pathways, such as glycolysis, TCA cycle, and beta-oxidation are present at higher levels in non-hormone-dependent breast cancer and triple-negative breast cancer than in hormone-dependent breast cancer, which correlates with breast cancer aggressiveness [13]. Metabolites of secondary bile acid metabolism, amino acid degradation, short-chain fatty acid production, and deconjugated hormones have also been shown to predict cancer aggressiveness [14,15,16]. 

The aim of our study was to identify metabolomic biomarkers specific to high-grade SBR in early-stage breast cancer. After identifying a reliable metabolomic signature, metabolic pathway analyses were performed.

## 2. Materials and Methods

### 2.1. Population

The training population consisted of 51 patients treated at our institution (Centre Antoine Lacassagne, Cancer Center of Nice) between March 2013 and September 2016 for a clinical stage I to III_B_ biopsy-proven breast cancer with an indication for adjuvant therapy after surgery. The validation population consisted of 49 patients treated in another institution (Centre Georges-François Leclerc, Cancer Center of Dijon) between February 2007 and July 2012 for a clinical stage II_A_ to IV biopsy-proven BC, with an indication for neoadjuvant therapy before surgery. All patients were included retrospectively in the study. The biopsy and tumor resection samples were quick-frozen and stored in the tumor biobanks of our respective facilities. All patients were treated according to current guidelines, with sequential chemotherapy including anthracyclines (epirubicin and cyclophosphamide) and taxanes before or after surgery and radiotherapy. HER2-positive status was defined as IHC3+ or IHC2+/FISH+. Patients with HER2-positive tumors were treated with trastuzumab and taxanes simultaneously for one year (total duration). Patients with luminal BC were then treated by endocrine therapy with Tamoxifen or an aromatase inhibitor, based on menopausal status.

### 2.2. Patient Data Collection and Statistical Analysis

Clinical, histological, radiological, and therapeutic data were retrospectively extracted from our facility’s digital records or collected by a clinical data monitor, including the SBR (Scarff–Bloom–Richardson) grade used to stratify breast cancer into low, intermediate, and high grades based on the nuclear grade, tubule formation, and mitotic rate [17,18]. Since the two study populations (training set and validation set) were different and to be able to extrapolate our results to real-life study populations, we analyzed and compared the clinical and tumor characteristics between the training set and the validation set using the *t*-student and Fisher’s exact test.

### 2.3. Sample Collection

Samples for the training set were collected during breast surgery. Samples for the validation set were collected during the diagnostic biopsy prior to neoadjuvant chemotherapy. All the samples were quickly deep-frozen and transferred to our facilities’ respective biobanks where they were stored at −80 °C until analysis. Samples from Dijon were transported to Nice at −80 °C prior to the metabolomic analysis. All samples were prepared and analyzed in the same facility.

### 2.4. Sample Preparation

Samples (50–100 mg tumor tissue or 20–40 mg biopsy sample) were placed in 1.5 mL Eppendorf tubes containing 1 mL of methanol, grinded manually with a piston and stored at −20 °C overnight. Samples were then centrifuged at 13,000 rpm for 15 min at 0 °C. Supernatants were transferred into new tubes and placed in a Speed Vac until complete liquid evaporation occurred. Samples were then stored at −80 °C until LC-MS analyses. They were resuspended in 100 µL of a 50% acetonitrile and 50% water mix before LC-MS analysis [19].

### 2.5. LC-MS Analysis

Liquid chromatography analysis was performed using a DIONEX Ultimate 3000 HPLC system (Thermo Fisher Scientific, Waltham, MA, USA). From each sample, 10 µL was injected onto a Synergi 4 µm Hydro-RP 80 Å, 250 × 3.0 mm column (Phenomenex, Le Pecq, France). The mobile phases were composed of 0.1% formic acid (Thermo Fisher Scientific) in water (A) and 0.1% formic acid in acetonitrile (B). The gradient was set as follows with a flow rate of 0.9 mL/min: 0% phase B from 0 to 5 min, 0–95% B from 5 to 21 min, holding at 95% B until 21.5 min, 95–0% B from 21.5 to 22 min, holding at 0% B until 25 min for column equilibration. Mass spectrometry analysis was carried out on a Q Exactive Plus Orbitrap mass spectrometer (Thermo Scientific, Waltham, MA, USA) with a heated electrospray ionization source, HESI II, operating in both positive and negative mode. High-resolution accurate-mass full-scan MS and the top 5 MS2 spectra were collected in a data-dependent fashion at a resolving power of 70,000 and 35,000 at *m*/*z* 400, respectively. This standard procedure has been described in more detail in the cited publications [20,21,22,23,24,25]. The analyses were performed separately on each of the two groups: the first group consisted of the 51 tumors of the training set and the second of the 49 tumors of the validation set.

### 2.6. Data Preprocessing and Metabolite Identification

The raw data obtained for the two groups in positive and negative ionization modes were analyzed separately with MzMine (Version 2.38) [26,27]. Individual chromatograms were built for each mass with a noise threshold of 10^5^. A local minimum search algorithm was used to select the validated peaks. Peaks were then aligned by RANSAC (random sample consensus) algorithm with a tolerance of 10 ppm in *m*/*z* and 1 min retention time. Missing values were filled, as far as possible, with the same *m*/*z* and RT range as observed for detected samples, using the gap-filling tool. Peaks were then predicted using the Human Metabolome DataBase (HMDB, version 3.0) by searching for M + H^+^ and M − H^+^ ion forms in positive and negative modes, respectively, with a mass tolerance of 15 ppm. Only predicted peaks were included in the final analysis. A linear normalization was performed using the average intensity of each sample as a normalization factor. Only metabolites with no null values after pre-processing were selected for final analysis. If a metabolite was detected in both positive and negative modes, only the mode with the highest average intensity was considered. Finally, a filtering function was applied before statistical analysis selecting only the metabolites with the highest average intensity. This step allowed us to eliminate metabolites that could be considered as background signals or for which quantification was not robust enough.

### 2.7. Metabolite Selection

The metabolite selection methodology was established as follows to ensure the reproducibility of the analyses. Since the two raw databases (the training set and the validation set) had been merged, only common predicted metabolites were kept. Data were filtered for correlated metabolites, signal intensity, isotope, duplicates, artifacts, and drugs. Metabolite validations were performed with MS2 (from MZmine and/or using Compound Discoverer analysis). MS2 matches of the first 25 metabolites of interest (top list of the statistical analysis) are available in the Supplementary Materials (ms2.xls). The final table with all metabolites is available in the “HMDBval_PLSNice” sheet of the “MS2” Excel file (ms2.xls Supplementary Materials).

### 2.8. Statistical and Pathway Analyses

All statistical analyses were performed online using MetaboAnalyst (https://www.metaboanalyst.ca/, accessed on 21 December 2022) version 5.0 [28]. The only sample normalization, data transformation, and data scaling method used was the log transformation. Sum or median sample normalizations did not improve the performance of the chemometrics analysis (Principal Component Analysis or PCA; Partial Least Squares Discriminant Analysis or PLS-DA). PLS-DA analysis was used to establish score plots, loading plots, and cross validations (performance accuracy, R2, Q2). Receiver Operating Characteristic (ROC) curves, heatmap graphs, exploration of metabolite set enrichment, and metabolic pathway analyses were generated online using MetaboAnalyst (https://www.metaboanalyst.ca/, accessed on 21 December 2022). The tryptophan pathway was interpreted using data from the SMP and Kegg pathway.

## 3. Results

### 3.1. Clinical and Tumor Characteristics

Fifty-one patients were analyzed in the training set and 49 patients in the validation set. Clinical and tumor characteristics are described in Table 1. Median ages were statistically different (*p* < 0.00001) with 65 years (range: 37–88) for the training set and 51 years (range: 26–70) for the validation set. Tumor size, T stage, and N stage also differed statistically with more unfavorable tumor characteristics in the validation set compared to the training set: median tumor size 40 mm, 10.2% of T4, 71.4% of axillary lymph node invasion vs. median tumor size 30 mm, 1.9% of T4, 47.1% of axillary lymph node invasion. These differences could be explained by locally advanced and localized settings. However, the cellular characteristics of the two groups were comparable: the main histological feature was invasive ductal carcinoma (82.5% and 91.8%), almost half of the patients had SBR grade 3 tumors in both populations, and no statistical differences were observed for Ki67, estrogen-receptor, progesterone-receptor, and HER2-receptor status. Despite clinically different study populations, these two groups could therefore be used to analyze intra-tumor cellular aggressiveness.

### 3.2. SBR Grade Metabolomic Signature Discriminated between High-Grade (Grade III) and Low-Grade (Grade I–II) Groups

**Figure 1:**
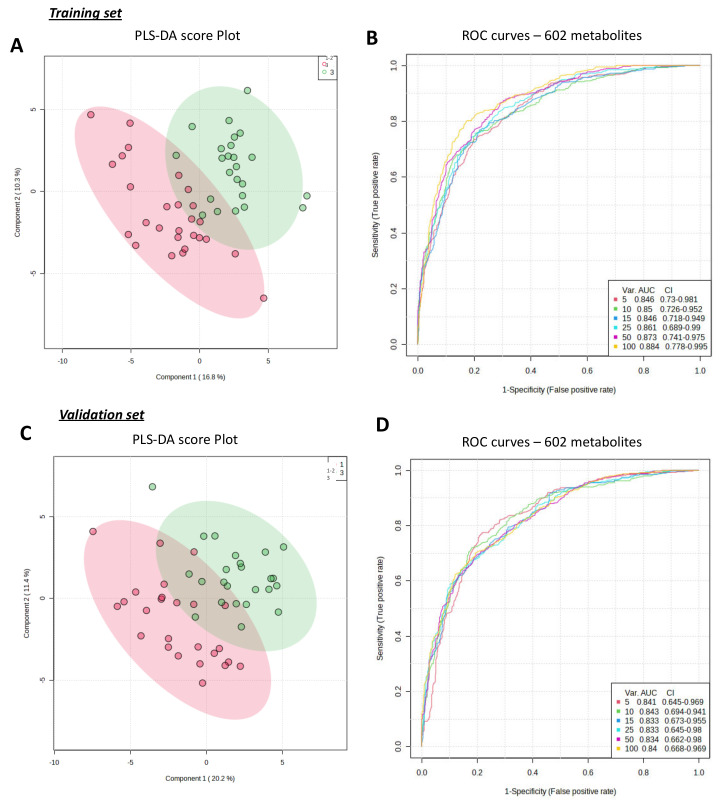
Metabolomic fingerprinting allowed accurate discrimination of SBR grades using 602 predicted metabolites found in both the training set and the validation set. (**A**) shows the score plot of the PLS-DA on the training set, which can accurately discriminate between high-grade (grade III—green dots) and low-grade (grade I–II—red dots) groups. (**B**) shows the AUCs of the ROC of different metabolomic signatures for the training set, which included an increasing number of metabolites (var.), with their respective 95% confidence interval values (95%CI). The score plot of the PLS-DA and ROC curves for the validation set are shown in (**C**,**D**), respectively.

The metabolome from samples collected during breast surgery (training set) and those collected during diagnostic biopsy (validation set) were analyzed by liquid chromatography coupled with mass spectrometry (LC-MS) according to our standard procedures [20,22,29]. Posttreatment of the obtained data generated a database of 602 predicted metabolites. Peak intensities of these predicted metabolites in the 100 tumor samples are included in the Supplementary Materials (training_set.csv and validation_set.csv). Patients in both groups were classified as high-grade (grade III) or low-grade (grade I-II) according to their clinical characteristics. Principal Component Analyses (PCA) performed with MetaboAnalyst showed that the two groups could not be distinguished with this unsupervised method (score plots illustrated in Figure S1A,B). Supervised analyses were subsequently performed on the two cohorts independently. For the training set, the best PLS-DA model was obtained for three components with an accuracy value of 0.79, R2 = 0.84 and Q2 = 0.38 (Figure 1A,B, values illustrated in Figure S1C–E). For the validation set, the best model was obtained for two components with an accuracy value of 0.78, R2 = 0.69 and Q2 = 0.38 (Figure 1C,D, values illustrated in Figure S1D–F). Multivariate Receiver Operating Characteristic (ROC) curve analyses were also performed using MetaboAnalyst. Areas Under the Curves (AUCs) reached 0.884 (CI95% 0.778–0.995) for the training set and 0.84 (CI95% 0.668–0.969) for the validation set. 

**Figure 2:**
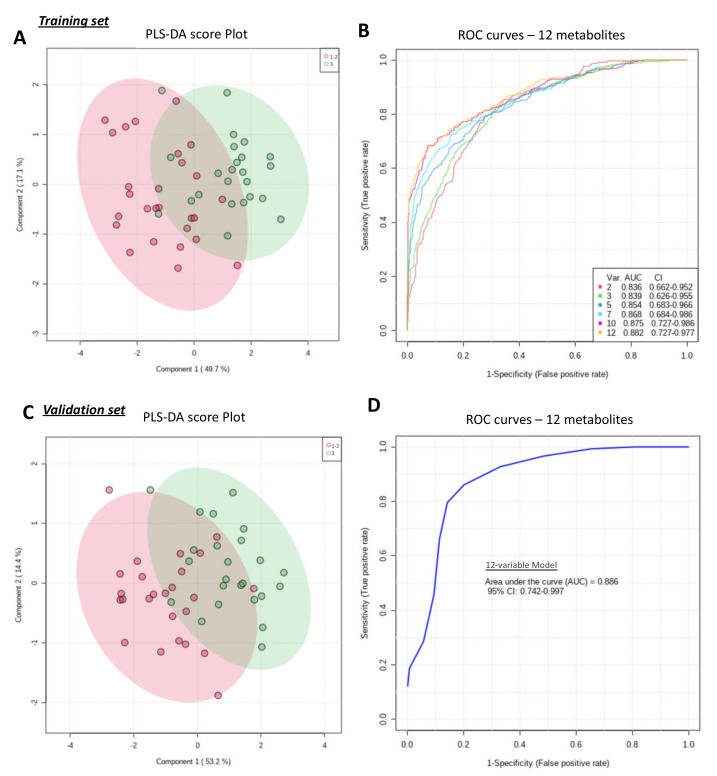
Metabolomic fingerprinting allowed accurate discrimination of SBR grades using the top 12 most important metabolites. The top 12 metabolites (N1,N12-Diacetylspermine, N’Formylkynurenine, N-(1-Deoxy-1-fructosyl)phenyalanine, fructoseglycine, malonylcarnitine, L-L-Homoglutathione, 5-Hydroxy-L-tryptophan, 8-Methpxykynurenate, L-Dopa, L-Kynurenine, N-Acetylproline and 5-Hydroxyindoleacetic acid) were determined from previous Partial Least Squares Discriminant Analyses (PLS-DA—see Figure 1A). (**A**) shows the score plot of the PLS-DA for the training set, which accurately discriminates between high-grade (grade III—green dots) and low-grade (grade I–II—red dots) groups. (**B**) shows the AUCs of the ROC of different metabolomic signatures in the training set, which included an increasing number of metabolites (var.), with their respective 95% confidence interval values (95%CI). The score plot of the PLS-DA and ROC curves for the validation set are shown in (**C**,**D**), respectively.

Score plots of PLS-DA analyses using the top 12 metabolites are illustrated in Figure 2A–C. The best models were obtained with two components. After cross-validation, the accuracy values were 0.81 and 0.82, R2 scores were 0.57 and 0.55, and Q2 scores were 0.44431 and 0.40147 for the training set and the validation set, respectively (Figure S2). AUC or ROC curves were 0.882 (CI95% 0.727–0.977) for the training set and 0.886 (CI95% 0.742–0.997) for the validation set (Figure 2B–D). The performance of the grade SBR metabolomic signature could not be improved by either sample normalization (Figure S3) or by increasing the number of metabolites included up to 25 (Figure S4). The top 12 metabolites as well as the top 25 were validated using MS2 matches (for details see Supplementary Materials).

### 3.3. PLS-DA Models Identified a Discriminatory Signature with the Top 12 Metabolites 

**Figure 3:**
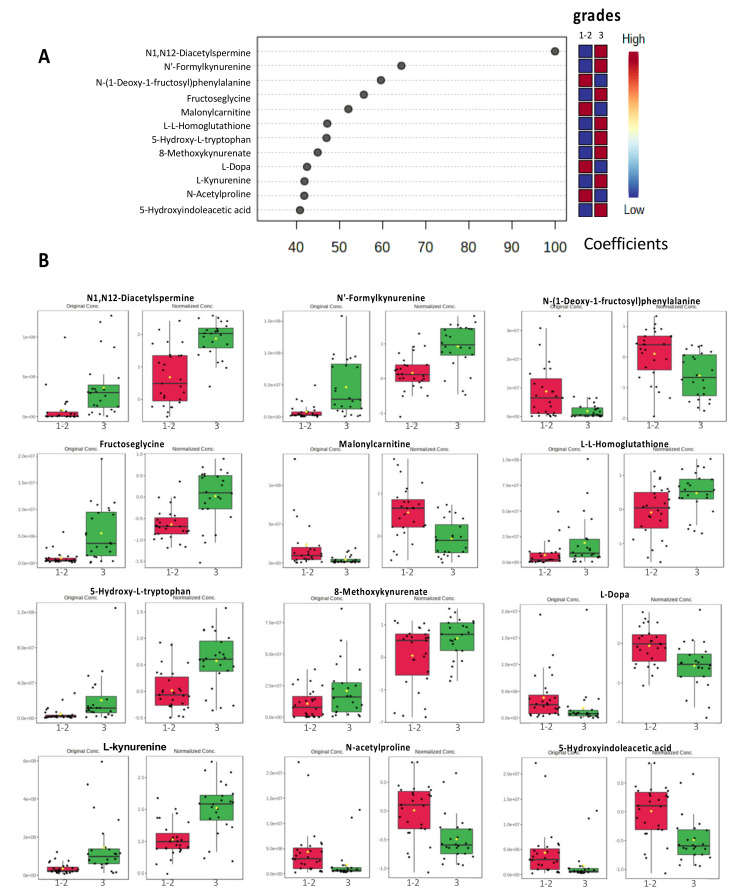
Importance and variation of the top 12 most important metabolites. (**A**) shows the coefficient score plot for the top 12 most important metabolite features identified by PLS-DA. In the right column, the relative concentration of the metabolite is represented in blue when reduced or in red when increased. (**B**) Box plots illustrate the relative concentration of the top 12 most important metabolite features identified by PLS-DA in high-grade (grade III—green boxes) and low-grade (grade I-II—red boxes) groups. The exact names of the metabolites were verified by matching experimental MS2 results with MS2 databases (HMDB).

The top 12 metabolites that provide a putative discriminatory signature are shown according to their coefficient scores in Figure 3. These 12 most relevant metabolites were N1,N12-Diacetylspermine (coefficient score = 100), N’Formylkynurenine (coefficient 65.7), N-(1-Deoxy-1-fructosyl)phenyalanine (coefficient 57.3), fructoseglycine (coefficient 53.8), malonylcarnitine (coefficient 49.1), L-L-Homoglutathione (coefficient 48.9), 5-Hydroxy-L-tryptophan (coefficient 46.9), 8-Methpxykynurenate (coefficient 46.5), L-Dopa (coefficient 44.0), L-Kynurenine (coefficient 43.0), N-Acetylproline (coefficient 39.6), and 5-Hydroxyindoleacetic acid (coefficient 39.0). 

### 3.4. Metabolic Pathway Analysis

**Figure 4:**
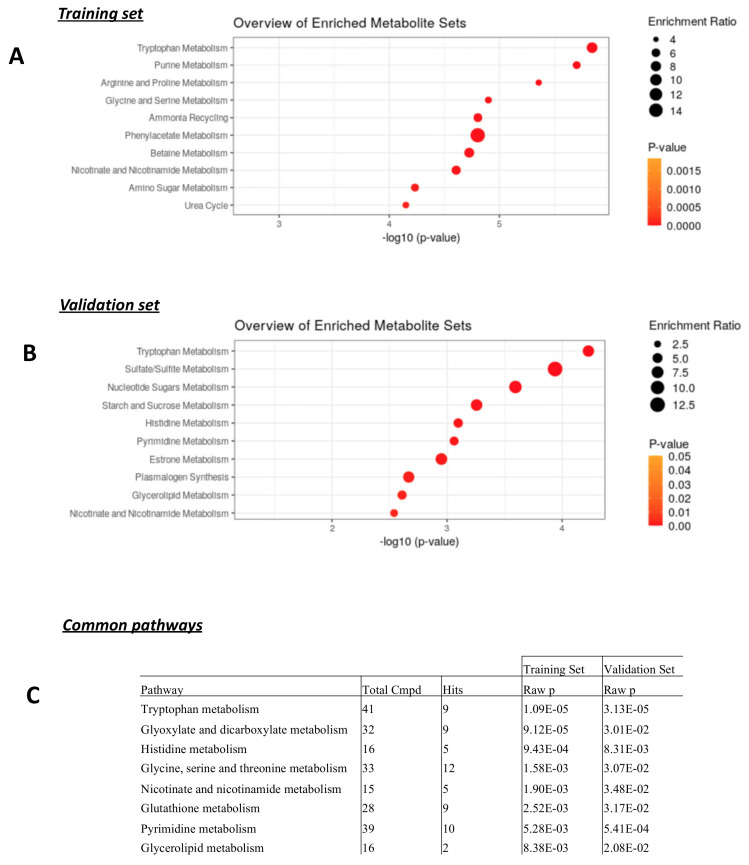
Metabolite set enrichment analyses and metabolic pathway analyses highlighted the tryptophan metabolism. The top 10 enriched metabolite sets in the analyses performed on the training set and the validation set are shown in (**A**,**B**), respectively. Metabolic pathway analyses were also performed on both sets and the top 7 common significant metabolic pathways are illustrated in (**C**). More details are provided in Table S1.

Metabolite set enrichment analyses were performed separately on the training set and the validation set. For both sets, the most significant metabolic pathway (*p*-value < 0.0005) with an enrichment ratio of eight was the tryptophan pathway (the top 10 enrichments are shown in Figure 4A,B for both the training and the validation set). A similar result was obtained with metabolic pathway analyses (the top seven common pathways are shown in Figure 4C, more details are shown in Table S1). The most relevant common pathway between the training set and the validation set was the tryptophan metabolism pathway with 9 hits and *p*-values < 0.00005 (training set: *p* = 1.09 × 10^−5^, validation set: *p* = 3.13 × 10^−5^ Figure 4C). The matched metabolites of the tryptophan metabolism pathway were N-Acetylserotonin, 5-Hydroxyindoleacetate, 5-Hydroxy-L-tryptophan, 3-Hydroxyanthranilate, L-Kynurenine, Indole-3-acetaldehyde, Formyl-N-acetyl-5-methoxykynurenamine, Cinnavalininate, and 4-(2-Amino-3-hydroxyphenyl)-2,4-dioxobutanoate. 

**Figure 5:**
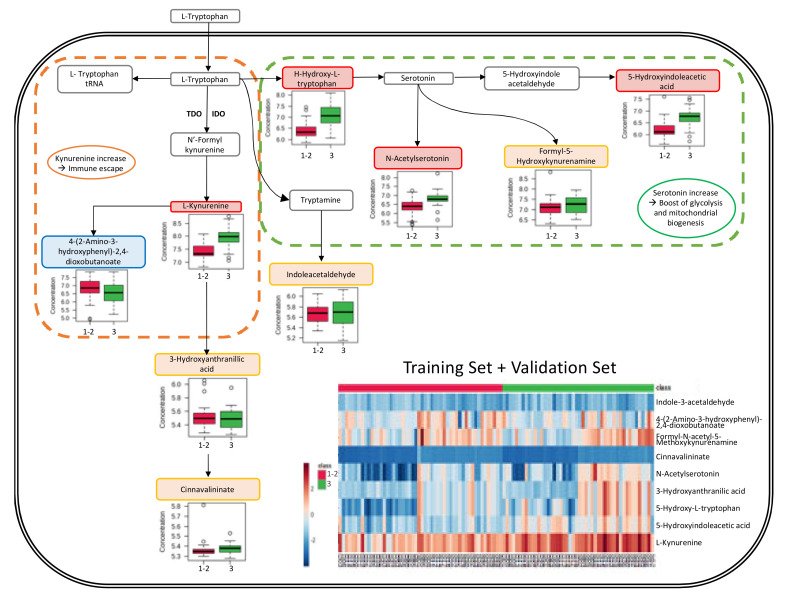
Schematic representation of metabolic pathway changes. The tryptophan pathway illustrations have been adapted from the Small Molecule Pathway database (https://www.smpdb.ca/view/SMP0000063, accessed on 21 December 2022). Box plots illustrate the relative concentration of the main tryptophan catabolites in high-grade (grade III—green boxes) and low-grade (grade I–II—red boxes) groups. Metabolite names are shown in colored boxes: red boxes relate to higher concentrations in high-grade samples; orange boxes to equivalent concentrations in high-grade and low-grade samples; green boxes to lower concentrations in high-grade samples. Heatmap representations of relative concentrations of tryptophan catabolites are shown for all samples (together for the training set and the validation set). Results of the low-grade (grade I–II—red labels on the top line) group are positioned in the left part of the heatmap and those of the high-grade (grade III—green labels) group in the right part.

The analysis of the tryptophan pathway using the KEGG pathway database (Kyoto Encyclopedia of Genes and Genomes (https://www.genome.jp/kegg/pathway.html, accessed on 21 December 2022) (Figure S5) and the SMP database (Small Molecule Pathway) (https://www.smpdb.ca/, accessed on 21 December 2022) revealed an activation of the aromatic amino acid metabolism and serotonin metabolism pathways with a noticeable increase of L-Kynurenine, 5-Hydroxy-L-tryptophan, N-acetylserotonin, and 5-Hydroxyindolacetate in high-grade tumors (results are shown in Figure 5, see also Figure S6). The relative metabolite levels are also presented in a heatmap revealing the considerable variation in metabolite levels between the different samples (included in Figure 5, see also Figure S7).

## 4. Discussion

This study is the first to analyze the metabolomic profiles of high-grade tumors regardless of their histologic subtype. We identified a metabolic signature for high-grade tumors and obtained AUCs for the training and the validation set above 0.88, showing that our model discriminates high-grade from low-grade tumors with a probability of almost 90%. This signature is not intended to replace the classification system currently used in clinical practice, but it does provide a better analysis of the underlying cellular signaling pathways.

To date, only a few studies have been published on the metabolomic signatures of high-grade SBR. In a study of 139 serum samples from grades I, II, III breast cancer patients and 155 healthy volunteers, Hadi NI et al. [30] showed that the increased levels of glucopyranoside, tetradecane, mannose, and benzene 1,2-dicarboxylic acid allow a differentiation between the various grades. Despite their encouraging results, the authors concluded that a larger sample was needed to further support their findings and to define the metabolic differences between tumor grades more precisely [30]. However, a comparison with our results is not possible because Hadi’s group analyzed serum samples while we worked on tissue samples. In addition, Hadi and her colleagues performed gas chromatography analyses coupled with a mass spectrometer (GC-MS), while we performed LC-MS analyses, which may lead to the identification of different metabolites.

### 4.1. Strengths and Weaknesses of the Study

We have already performed several studies using similar experimental procedures and have shown that it reliably identifies many metabolites. Despite the use of only one method (LC-MS), this study allowed us to identify and evaluate a large number of metabolites in only small amounts of tumor tissue using biopsy samples (validation set) and comparing them with larger samples from breast surgery (training set). One of the main strengths of our study is that it was conducted on two different sample cohorts from two different patient groups (i.e., biopsies of locally advanced tumors collected from patients in the Dijon aera for the validation set and breast surgery samples of localized tumors on from patients in the Nice aera for the training set). Furthermore, the samples were analyzed in two separate and independent runs (first, the 51 tumors from the training set and second, the 49 tumors from the validation set). This could have led to the statistically significant differences observed in clinical and tumor characteristics, but it also allowed to detect only large differences and identify only robust signatures.

In the present study, metabolic analyses were performed on breast tumor tissue only. No analysis was performed on peripheral blood samples. Since the metabolite signature identified in breast tumor tissue cannot be extrapolated to the signature expected in peripheral blood, it is not suitable for the early detection of tumors in clinical routine. However, in the case of primary surgical treatment, metabolomic analysis of tumor resection samples allows, for example, the prediction of the occurrence of immunosuppression (and thus provides information about the efficiency of a potential immunotherapy).

### 4.2. N1,N12-Diacetylspermine Metabolite (DiAcSpm)

In our SBR signature, the most relevant metabolite was N1,N12-Diacetylspermine, an alkylamine with multiple amino groups (polyamine). In both sample sets, higher levels of N1,N12-Diacetylspermine were found in high-grade tumor samples than in samples from low-grade tumors (Figure 3). Polyamines are produced during cell division. They are then acetylated in the liver and finally excreted in the urine [31]. MYC is an oncogenic driver of tumor development, progression, and immune-suppression in triple-negative breast cancer (TNBC) [32,33,34,35]. A downstream target of MYC is ornithine decarboxylase (ODC), a rate-limiting enzyme of the polyamine metabolism [36,37]. Polyamines have been described to play a functional role in promoting neoplastic transformation and growth [38,39]. Among polyamine derivatives, N1,N12-diacetylspermines have recently attracted much attention in oncology, and urinary diacetylspermines have been described as highly sensitive tumor markers in many cancers, including breast cancer [31,40,41,42]. Previous studies have shown that high levels of acetylated polyamines are found in breast cancer in association with a simultaneous increase in spermidine and spermine N1 acetyltransferase (SAT1) activity and decreased polyamine oxidase activity [43]. A functional study investigated the effects of spermine on the estrogen receptor (ER) [44]. The obtained results suggest that spermine plays an important role in the regulation of ER ligand-binding and gene activation and thus also in hormone resistance. DiAcSpm was studied by Fahrmann et al. in triple negative breast cancer (TNBC) patients [45]. Serum samples from TNBC patients showed a higher DiAcSpm level than samples from non-TNBC patients and healthy volunteers. In addition, Fahrmann et al. provided evidence that elevated plasma DiAcSpm levels are associated with low immune infiltrate, reduced immune-related gene signatures, early recurrence (<1 year), worse 5-year distant metastasis-free survival and 5-year overall survival. Here, we report the increase of DiAcSpm in breast tissue from low- and high-grade tumors, regardless of their histological subtype.

### 4.3. Kynurenine Synthesis via the Tryptophan Pathway

The kynurenine to tryptophan catabolism is a known mechanism involved in the modulation of the immune system and has been extensively studied in cancer (33). Tryptophan is converted to kynurenine by indoleamine 2,3-dioxygenase 1 (IDO1), its splice variant IDO2 and tryptophan 2,3-dioxygenase(TDO) [46]. IDO1 is a key factor in maintaining immune tolerance [47]. Its expression increases in response to several inflammatory cytokines, such as interferon-γ, which acts as an endogenous mechanism to prevent an excessive immune response [48]. IDO1 is expressed in multiple tumor types and is associated with reduced activation of cytotoxic cells, increased infiltration of tumor-regulating T-cells, poorer survival rates [49,50,51,52,53,54,55], and increased drug resistance [56,57,58,59]. Wei et al. [60] measured IDO1 expression in paraffin-embedded breast cancer tissue samples. The group found that IDO is expressed in 64% of the samples. D’Amato et al. [61] suggested an important role for TDO in aggressive breast cancer subtypes, as high TDO levels were found in primary breast tumors associated with shorter overall survival.

Several molecular mechanisms have been described to explain how IDO contributes to tumor-induced tolerance [62,63,64,65,66]. IDO promotes, for example, the formation of immunosuppressive antigen presenting cells (APCs). Furthermore, overexpression of IDO1 in APCs activates the kynurenine pathway, which facilitates kynurenine release and tryptophan consumption. Tryptophan catabolites (kynurenine and its downstream metabolites) operate by activating the aryl hydrocarbon receptor involved in the immune response. Consumption of tryptophan leads to the activation of GCN2 and inhibition of mTOR, which in turn is responsible for Treg differentiation, MDSCS activation and inhibition of T-lymphocytes and natural killer cells [62,63,64,65,66]. In our study, both metabolite-set enrichment analyses and metabolic pathway analyses showed that the tryptophan pathway is more strongly activated in high-grade tumors than in low-grade tumors. The SBR signature of the 12 major metabolites revealed increased levels for 4 tryptophan catabolites (N’-formylkynurenine, 5-hydroxy-L-tryptophan, 8-methoxykynurenate, and L-kynurenine) (Figure 3) in high-grade tumors compared with low-grade tumors. Using breast cancer tissue provided by Duke University Medical Center, Tang et al. showed that kynurenine levels are significantly higher in ER-negative tumors than in ER-positive tumors [67]. Here, we report the first results that associate high-grade tumors with the tryptophan pathway in breast cancer regardless of the histological subtype. 

### 4.4. Serotonin Implications

Our results also suggest a greater activation of the serotonin pathway in high-grade tumors. Although serotonin is mainly known as a neurotransmitter, it is also synthesized by epithelial cells in the mammary gland by tryptophan hydroxylase 1 (TPH1) and plays a role in regulating epithelial homeostasis in breast cancers. Serotonin may produce multiple effects through interaction with a variety of receptors involved in different signaling pathways [68]. The alteration of serotonin and serotonin receptor expression patterns leads to dysregulation of epithelial homeostasis, which has been associated with the initial events of breast cancer development, tumorigenesis and tumor progression [69,70,71,72]. Tumors can down-regulate enzymes of serotonin synthesis, decreasing the consumption of tryptophan by the serotonin pathway to increase the consumption of tryptophan by the tryptophan/kynurenine pathway [73]. However, in our study, levels of metabolites of the serotonin pathway were also higher with an increase of N-Acetylserotonin and 5-Hydroxyindoleacetic acid. The serotonin pathway could therefore be involved in tumor aggressiveness in breast cancer independently of the tryptophan/kynurenine pathway. Serotonin has already been shown to affect the proliferation and metabolism of breast cancer cells by triggering two distinct signaling pathways: Jak1/STAT3 which boosts glycolysis by upregulating PKM2, and adenylyl cyclase/PKA which promotes mitochondrial biogenesis [74]. In addition, several studies have suggested that the expression of serotonin and its receptors in immune cells can modulate the immune response, especially in the case of inflammation [75,76]. Other studies have indicated that the immune effects of serotonin include the suppression of IL-1β and TNF-α release in peripheral blood cells and the activation of T-cells [77]. Here we identified high levels of metabolites of the serotonin pathway in high-grade patients. This finding suggests that the serotonin pathway is involved in the aggressiveness and immunosuppression of high-grade breast cancer.

### 4.5. Grade and Immune Response

Our study showed that high-grade tumors are related to higher levels of DiAcSp and tryptophan-derived metabolites, both of which are involved in the immune response through Treg differentiation, T cell and natural killer cell inhibition. These findings raise the question of whether the aggressiveness of high-grade tumors could depend on immune escape. Other studies have already indicated that T cells play an essential role in limiting tumor development and that in breast cancer, CD4+ and CD8+ infiltrating T cells are abundant in high-grade ductal carcinoma in situ as well as in invasive carcinoma [78,79,80]. Higher T_reg_infiltration is associated with high grade but not with tumor subtype, size of the invasive tumor, lymph node status, or disease stage [81].

Considering these immune escape mechanisms, targeting spermine and tryptophan metabolism could decrease Treg differentiation and reactivate T cells and natural killer cells, thereby reducing immune escape and restoring anti-tumoral immunity. Moreover, targeting both spermine and tryptophan metabolism could create a synergistic effect. Several strategies have been outlined by Peyraud et al. including three different strategies that target the IDO/TDO-Kyn-AhR signaling circuit in cancer treatment: (i) pharmacological inhibition of IDO/TDO by IDO inhibitors, (ii), systemic depletion of Kyn by engineered kynureninase, and (iii) blockade of AhR activation by synthetic AhR modulators [82] (Table 2). To date, no study has been able to demonstrate the benefit of these targeted therapies.

Interestingly, our study showed that the activation of the tryptophan pathway was not homogenous among all high-grade patients. Indeed, the L-Kynurenine levels were not high in the analyzed samples from high-grade patients (Figure 5), which may have an impact on the efficacy of the targeted therapies tested. Better selection of targeted therapies for each candidate using previous metabolomic assays may improve efficacy. One should note that, after determination of discriminant biomarkers, accessibility of the targeted metabolomic technique is a major element of applicability in routine care. Such putative personalized medicine will be analyzed in further studies. Finally, with the advent of immunotherapy in neo-adjuvant [83] and first-line metastatic [84] triple-negative breast cancer, the theragnostic value of the activation of these metabolic pathways may be analyzed in the future.s

## 5. Conclusions

Here, we report the identification of a metabolic signature for high-grade invasive tumors with AUCs greater than 0.88 on both the training set and the validation set, suggesting that the model has a nearly 90% chance of being able to distinguish high-grade from low-grade tumors. This may be of interest in cases of heterogeneity but essentially confirms the performance of the metabolomic analysis. Our results showed that high-grade invasive tumors are related to the metabolism of DiAcSp and tryptophan, both involved in the inhibition of the immune response. Targeting these pathways could restore anti-tumor immunity or activate immunogenicity and create a synergistic effect with immunotherapy. Although the efficacy of this strategy has not been demonstrated in recent studies, metabolic analysis may allow better selection of the most appropriate therapy for each patient. Personalized immunotherapy using theragnostic metabolomic signatures needs to be evaluated in further studies.

## Figures and Tables

**Table 1:** Clinical and tumor characteristics (training set and validation set).

		Training Set		Validation Set		
		(*n* = 51)		(*n* = 49)		
		N/med	(%/SD)	N/med	(%/SD)	*p*
Age						*p* < 0.00001 (£)
	median	65		51		
	min-max	37–88		26–70		
Histology						NS ($)
	DIC	48	(82.5%)	45	(91.8%)	
	LIC	3	(12.5%)	3	(6.1%)	
	other	0	(0.0%)	1	(2.0%)	
Tumor size (mm)		30 *	(21.9)	40 **	(22.4)	*p* < 0.00001 (£)
T						*p* = 0.001 ($)
	T1	13	(25.5%)	3	(6.1%)	
	T2	26	(51.0%)	37	(75.5%)	
	T3	11	(21.6%)	3	(6.1%)	
	T4	1	(1.9%)	5	(10.2%)	
	unknown	0	(0.0%)	1	(2.0%)	
N						*p* = 0.002 ($)
	N0	26	(51.0%)	14	(28.6%)	
	N1	18	(35.3%)	34	(69.4%)	
	N2	3	(5.9%)	1	(2.0%)	
	N3	3	(5.9%)	0	(0.0%)	
	unknown	1	(1.9%)	0	(0.0%)	
SBR grading						NS ($)
	I	5	(9.8%)	5	(10.2%)	
	II	22	(43.1%)	20	(40.8%)	
	III	24	(47.1%)	24	(50.0%)	
Ki67%						NS (£)
	median	35	(29.3)	60	(23.0)	
	≤10%	4	(7.8%)	1	(2.0%)	
Estrogen-receptor						NS (£/$)
	Mean	50.2	(47.9)	65.4	(43.6)	
	≥10% of cells	29	(56.9%)	28	(57.1%)	
Progesteron-receptor						NS (£/$)
	Mean	40.3	(42.5)	43.4	(38.3)	
	≥10% of cells	28	(54.9%)	31	(63.3%)	
HER2-positive receptor						NS ($)
	HER2 not amplified	40	(78.4%)	41	(83.7%)	
	HER2 amplified	11	(21.6%)	8	(16.3%)	

Data retrospectively extracted from digital records or collected by a clinical data monitor. DIC: Ductal Invasive Carcinoma; pT: primary tumor (TNM); pN: regional lymph nodes (TNM); SBR: Scarff–Bloom and Richardson; med: median; SD: standard deviation. * Size assessed on excisional specimen (*n* = 52). ** Size assessed on ultrasound mammography (*n* = 48). (£) *t*-student test. ($) Fisher’s exact test. NS: not statistically significant.

**Table 2:** Clinical trials targeting the IDO/TDO-Kyn-AhR signaling. Past and recruiting trials, adapted from Peyraud et al. [82] IDO: indoleamine 2,3-dioxygenase; TDO: Tryptophan 2,3-dioxygenase; TNBC: triple-negative breast cancer; BID: twice daily; Q3W: every 3 weeks; ORR: objective response rate; DCR: disease control rate; PR: partial response; SD:s table disease; QD: daily; PD1: programmed cell death protein 1; PD-L1: programmed death-ligand 1; Kyn: kynurenine; AhR: aryl hydrocarbon receptor.

NCT Number	Phase	Number of Patients	Trial Title	Intervention	Main Results
**Pharmacological Inhibition of IDO-TDO/IDO Inhibitor**					
NCT02178722	I/II	3 TNBC	Study to explore the safety, tolerability and efficacy of MK-3475 combined with INCB024360 in participants with selected cancers	Epacadostat 1 BID combined with pembrolizumab Q3W	Acceptable safety profile TNBC: ORR 10%; DCR 36%
NCT02471846	I	25 (17 TNBC)	A study of GDC-0919 and atezolizumab combination treatment in participants with locally advanced or metastatic solid tumors	Navoximod BID combined with atezolizumab Q3W	Advanced cancer: PR 9%; ORR 10%, SD 24%; Decreasing plasma Kyn with increasing doses
NCT02658890	I/II	627 advanced cancer	An investigational immuno-therapy study of BMS-986205 given combined with nivolumab and combined with both nivolumab and ipilimumab in cancers that are advanced or have spread	Linrodostat combined with immunotherapy (nivolumab or nivolumab+ipilimumab)	Acceptable safety profile No efficicacy results yet
NCT03343613	I	90 advanced cancer	A study of LY3381916 alone or combined with LY3300054 in participants with solid tumors	LY3381916 QD combined with LY3300054 (anti-PD-L1) Q2W	Best response: SD
NCT03328026	I/II	60 breast cancer	Study of SV-BR-1-GM combined with retifanlimab	Epacadostat + Retifanlimab (anti-PD1) + SV-BR-1-GM (vaccine)	Recruiting
**Systemic depletion of Kyn/Kynureninase**					
-	-	-	-	-	-
**Blockade of AhR activation / synthetic AhR modulator**					
NCT04200963	I	93 advanced cancer	A phase 1a/b study of IK-175 as a single agent and combined with nivolumab in patients with locally advanced or metastatic solid tumors and urothelial carcinoma	IK-175 combined with nivolumab	Recruiting

## Data Availability

Peak intensities of theses predicted metabolites in the 100 tumor samples are provided as Supplementary Materials (training_set.csv and validation_set.csv). MS2 matches of the first 25 metabolites of interest (top list of the statistical analysis) are available in the Supplementary Materials (ms2.xls).

## Supplementary Materials

The following supporting information can be downloaded at: https://www.mdpi.com/article/10.3390/cancers15071941/s1, Figure S1: Untargeted metabolomic statistical analyses of SBR grades of the training set (*n* = 51) and the validation set (*n* = 49) using 602 metabolites; Figure S2: Untargeted metabolomic statistical analyses of SBR grades in the training set (*n* = 51) and the validation set (n = 49) using the top 12 metabolites; Figure S3: Metabolomic fingerprinting allowed accurate discrimination of SBR grades using the top 25 most important metabolites; Figure S4: Untargeted metabolomic statistical analyses of SBR grades of the training set and validation set using the top 25 metabolites; Figure S5: Schematic representation of metabolic pathway changes; Figure S6: Schematic representation of metabolic pathway changes; Figure S7: Heatmap representation of relative concentrations of tryptophan catabolites of the training set and the validation set; Table S1: Significant pathways of the SBR grade analysis.
